# Animal Models of Interferon Signature Positive Lupus

**DOI:** 10.3389/fimmu.2015.00291

**Published:** 2015-06-05

**Authors:** Haoyang Zhuang, Christopher Szeto, Shuhong Han, Lijun Yang, Westley H. Reeves

**Affiliations:** ^1^Division of Rheumatology and Clinical Immunology, Department of Medicine, University of Florida, Gainesville, FL, USA; ^2^Department of Pathology, Immunology, and Laboratory Medicine, University of Florida, Gainesville, FL, USA

**Keywords:** interferon alpha, lupus erythematosus, systemic, animal models, toll-like receptors, pristane, apoptosis, B-cell activation

## Abstract

Human lupus is strongly associated with a gene expression signature characterized by over-expression of Type I interferon-regulated genes. A strong interferon signature generally is not seen in the standard mouse models of lupus, despite considerable evidence for the involvement of toll-like receptor-driven interferon production. In contrast, pristane-induced lupus exhibits a prominent TLR7-dependent interferon signature. Importantly, genetic disorders with dysregulated interferon production in both human beings and mice cause severe autoinflammatory diseases but not the typical manifestations of lupus, suggesting that interferon over-production is insufficient to cause systemic lupus erythematosus itself. Single-gene models in mice suggest that lupus-like disease may result from abnormalities in B-cell activation and the clearance of dead cells. Pristane may mimic human systemic lupus erythematosus by causing synergistic abnormalities in interferon production along with defective clearance of apoptotic cells and over-active B-cell signaling.

Systemic lupus erythematosus (SLE) is defined by a complex clinical syndrome (e.g., arthritis, skin rashes, oral ulcers, serositis, glomerulonephritis, nervous system involvement) and the production of antinuclear antibodies (ANAs) such as anti-Sm and anti-double-stranded (ds) DNA (1, 2). It has become increasingly clear that over-production of Type I interferon (IFN-I) is strongly associated with SLE and that this cytokine is involved in disease pathogenesis. Over-expression of a group of interferon-stimulated genes (ISGs) by peripheral blood mononuclear cells from SLE patients is termed the interferon signature (3, 4). This gene expression program is seen in over two-thirds of adult and nearly all pediatric SLE patients who are defined by four or more of the ACR Classification Criteria (2). Adults exhibiting the interferon signature have an increased frequency of autoantibodies against dsDNA, Sm, RNP, and Ro, lower complement levels, and a higher frequency of lupus nephritis and possibly cutaneous manifestations than interferon signature negative patients (5–7).

Polymorphisms of a number of genes have been identified by genome-wide association studies (GWAS) to be linked to disease susceptibility in human beings, and some, such as IRF-5, are linked to the regulation of IFN-I production (8, 9). Additionally, therapeutic use of IFNα and trisomy of the Type I IFN gene cluster are associated with lupus-like autoimmunity in human beings (10, 11). However, despite the strong association of IFN-I over-production with SLE and evidence that the severity of lupus-like disease usually is attenuated in mice lacking the Type I interferon receptor (IFNAR) (12, 13), our understanding of the relationship of IFN-I to SLE pathogenesis is incomplete. Animal models may help address several questions, including: (1) Why do only a few patients (one of 987 in one study) treated with IFNα develop lupus (14)? (2) Why does IFNα induce lupus-like manifestations, whereas IFNβ generally does not (10)? (3) Why does treatment with neutralizing antibodies against IFNα (e.g., rontalizumab) lead to improvement of lupus in the interferon signature negative subset but not in the interferon signature high subset (15)? (4) Why do children with “interferonopathies,” who are identified by the presence of a strong interferon signature, develop vasculopathy, nervous system disease, and other inflammatory manifestations but only rarely lupus (16, 17)? (5) Why do Stimulator of IFN Genes (STING)-deficient lupus mice paradoxically develop more severe lupus-like disease than controls, despite the fact that human gain-of-function mutations are associated with inflammatory disease and a high interferon signature (18, 19)? (6) Why does IFN-I protect MRL/*lpr* mice from lupus, while exacerbating it in other strains (20)? (7) How is IFN-I linked to the production of lupus-specific autoantibodies?

## Interferon in Spontaneous Lupus Models

Several mouse strains develop spontaneous lupus-like disease, but they usually do not develop sufficient clinical/serological manifestations to meet the criteria used to classify human SLE (2) (Table 1). The most-studied strains are (NZB × NZW)F1 (NZB/W), NZM2410 and related strains (inbred derivatives of NZB/W selected for nephritis), MRL/*lpr*, and BXSB male mice, although this is not a complete list. Other strains, some with single-gene defects, develop more limited lupus-like disease (see below). It is probably not appropriate to equate the production of ANAs or anti-dsDNA autoantibodies, even in the presence of glomerular immune deposits (IgG/IgM or C3 staining), with what would be defined as “SLE” in human beings by standard clinical criteria (2). Nevertheless, partial lupus-like syndromes in mice may provide important insights into the pathogenesis of the individual manifestations of SLE and the spontaneous models have the advantage that as in human SLE, genetic susceptibility factors are important. However, since the candidate susceptibility loci identified in mice often do not appear to be major factors in human SLE, it is necessary to carefully relate these models to clinical subsets of human SLE. This review focuses on the suitability of mouse models for studying the interferon signature positive subset of SLE, which as noted above is characterized clinically by the production of specific autoantibodies with an increased prevalence of nephritis and possibly cutaneous manifestations.

**Table 1:** **Selected animal models of SLE**.

Animal model^a^	IFN signature	Anti-DNA	Anti-Sm/RNP	Clinical^b^	SLE criteria^c^
NZB/W, NZM2410	Weak	Yes	No	ANAs, severe LN	3
MRL/*lpr*	Absent	Yes	Yes	ANAs, severe LN, arthritis, skin rash?	6
MRL+/+	Absent	Low (late)	Yes (late)	ANAs	2
B6/*lpr*	Absent	No	No	ANAs	1
BXSB male	N/A	Yes	No	ANAs, severe LN	3
BAFF Tg	N/A	Yes	No	ANAs, LN	3
MerTK KO	N/A	Yes	No	ANAs, LN, arthritis	4
Pristane	Strong	Yes	Yes	ANAs, LN, arthritis, DAH, anemia, serositis	8

*^a^Tg, transgenic; KO, knockout; N/A, not available*.

*^b^ANAs, antinuclear antibodies; LN, lupus nephritis; DAH, diffuse alveolar hemorrhage*.

*^c^Number of the SLICC criteria for classification of SLE that are met (in human beings, four criteria are needed to define lupus with 95% certainty, at least one clinical and one laboratory criteria)*.

### NZB/W and NZM

NZB/W and NZM (NZM2410, NZM2328, others) mice are similar, developing severe glomerulonephritis and anti-dsDNA autoantibodies without other clinical or serological manifestations of lupus (21) (Table 1). Anti-Sm/RNP autoantibodies are absent. We have found that they exhibit a weak interferon signature at 6 months and but not at 2 months of age (Szeto, et al., manuscript in preparation). As in human beings, NZB/W lupus is exacerbated by IFN-I over-expression with accelerated anti-dsDNA autoantibody production, increased proteinuria, and worse glomerulonephritis (22). NZB mice develop severe IFNAR-dependent autoimmune hemolytic anemia but otherwise have only mild lupus-like manifestations (12). It is not known whether or not they exhibit an interferon signature.

### MRL+/+ and MRL/*lpr*

MRL+/+ develop late-onset, mild, lupus-like disease, manifested primarily by autoantibody production (Table 1). Deficiency of Fas (*lpr* mutation) greatly accelerates the onset of anti-Sm and anti-dsDNA/chromatin autoantibodies, as well as severe glomerulonephritis in MRL/*lpr* mice. MRL/*lpr* mice do not develop anti-RNP autoantibodies, although they are strongly anti-Sm positive. In contrast to NZB/W, MRL+/+ and MRL/*lpr* mice do not show evidence of IFN-I over-production at 2 or 6 months of age (Szeto, et al., manuscript in preparation). In sharp contrast to the IFN-dependent disease in NZB/W, IFNAR deficiency exacerbates autoantibodies and nephritis in MRL/*lpr* mice (20). Paradoxically, although lupus-like disease is very mild in B6/*lpr* mice, autoantibody production is attenuated by IFNAR deficiency (23). Thus, background genes unique to MRL vs. B6 may play a major role in determining the phenotype of lupus-like disease and its IFN-I dependence.

### BXSB Male

Although human and most spontaneous murine lupus is more common and frequently more severe in females, development of lupus in BXSB mice is limited to males (Table 1). This is due to the presence of two active copies of TLR7 (one on the X-chromosome and another on the Y-chromosome) (24, 25). Male BXSB mice develop anti-dsDNA and RNA autoantibodies with nephritis. Female mice with only one active copy of TLR7 are protected. Increasing the copy number of TLR7 in B6 mice also increases the production of anti-RNA autoantibodies (26). BXSB male mice produce high levels of anti-dsDNA antibodies and anti-RNA antibodies, but not anti-Sm/RNP. They also develop severe nephritis. It is not known whether they exhibit an interferon signature.

## Role of Endogenous TLR7 and TLR9 Ligands in IFN-I Production

The endosomal toll-like receptors (TLRs) TLR7 and TLR9 promote IFN-I production via a signaling pathway involving the adapter protein MyD88, kinases (IRAK1, IRAK4, TRAF6, TRAF3, and TAK1), and the transcription factor IRF7 (27). Although there is some flexibility in the optimal stimulatory sequences, TLR7/TLR8 and TLR9 are receptors for AU-rich single-stranded RNA and non-methylated cytosine-guanosine (CpG) motifs in DNA, respectively (28). Although they evolved as pattern recognition receptors for microbial nucleic acids, they can recognize endogenous nucleic acids, including U1 RNA (RNA component of U1 small ribonucleoproteins, recognized by anti-Sm/RNP autoantibodies) and certain endogenous DNA motifs (28). TLR7 and TLR9 are expressed in dendritic cell (DC) subsets (high levels in plasmacytoid dendritic cells, pDCs), macrophages, and B-cells (29, 30). In pDCs and macrophages, TLR7/TLR9 engagement strongly promotes IFN-I production. Conversely, TLR7 expression is strongly stimulated by IFN-I. In human beings as well as mice, TLR8 is not associated with immunopathology, possibly reflecting the fact that pDCs and B-cells do not express it (28).

Endosomal TLRs play a central role in autoimmunity in spontaneous lupus models. Although IFN-I production is not directly involved in the pathogenesis of lupus-like disease in MRL/*lpr* mice, anti-Sm autoantibodies are abolished in TLR7-deficient mice, whereas anti-dsDNA is TLR9-dependent (28, 31). Treatment with a dual TLR7/TLR9 inhibitor prevents disease progression in NZB/W mice, and there is genetic evidence that TLR7 is involved in human SLE (32, 33). The dependence of lupus-like disease in MRL/*lpr* mice on TLR7/TLR9, but not IFN-I, suggests that TLR signaling may have a role beyond the induction of IFN-I. Consistent with that possibility, TLR7/TLR9 signaling has B-cell-intrinsic effects on B-cell proliferation, terminal differentiation of memory B-cells, and spontaneous germinal center formation (34).

It has become increasingly clear that the TLR7/TLR9 ligands driving IFN-I production in SLE are derived from endogenous nucleic acids. There are numerous examples of lupus-like autoimmunity in mice with defective clearance of dead cells (35), though less evidence is available that the disease is IFN-I-dependent. Cells undergoing apoptosis expose phosphatidylserine (PtdSer) on the outer leaflet of the cell membrane (36). This is recognized by a variety of receptors mediating the rapid phagocytosis and degradation of dead cells before they can release endogenous nucleic acids capable of engaging TLR7/TLR9. PtdSer on apoptotic cells is bound by MFG-E8 (a secreted protein), serum proteins (protein S and Gas6), and complement component C1q. Phagocytosis of apoptotic cells coated by MFG-E8 is facilitated by the integrins α_v_β_3_ and α_v_β_5_, whereas uptake of cells coated with protein S or Gas6 is mediated by the “TAM receptors” *T*yro3, *A*xl, and *M*ertk (35), and uptake of C1q-opsonized cells is mediated by the scavenger receptor SCARF1 (37). This promotes the non-inflammatory clearance of dead cells and the suppression of TLR signaling. Deficiency of Mertk or other TAM receptors in mice causes lupus-like disease manifestations, such as nephritis, arthritis, and production of anti-dsDNA autoantibodies, but not anti-Sm/RNP (38) (Table 1). The phenotype is most severe in TAM triple knockout mice, but apparent in single and double knockout animals (39). C1q-deficient mice exhibit autoantibodies, glomerular accumulation of dead cells, and nephritis (40). Similarly, the risk of lupus in C1q-deficient human beings is 93%. There is considerable redundancy of the receptors utilized for clearing apoptotic cells. Although single-gene mutations frequently are associated with lupus-like phenomena, that is not always the case. For example, deficiency of the PtdSer receptor Tim-4 is not associated with lupus-like disease (41).

## Interferon in Human and Murine Single-Gene Lupus Models

Mice with deletion or over-expression of certain single genes develop limited lupus-like syndromes mediated in part by IFN-I production. These disorders are associated with a limited spectrum of autoantibodies, usually only anti-DNA/chromatin, and renal immune complex deposition with or without histological changes and proteinuria (Table 1).

### BAFF-Transgenic Mice

B-cell survival and maturation are enhanced by transgenic (Tg) expression of B-cell activating factor of the TNF family (BAFF, also known as BLyS), which activates NF-κB2 in B-cells (42). BAFF over-expression is seen in SLE patients as well as NZB/W and MRL/*lpr* mice (43). Moreover, BAFF inhibitors (TACI-Ig in mice, belimumab in human beings) reduce disease severity. Increased BAFF-TACI signaling decreases the stringency of negative selection of autoreactive B-cells in the periphery, promoting survival of autoreactive B-cells (44). BAFF-Tg mice produce high levels of anti-dsDNA and histone autoantibodies as well as rheumatoid factor, but not anti-Sm/RNP (45). Autoantibody production in these mice is T-cell independent but requires B-cell-intrinsic signals through the TLR/MyD88 pathway (45). TLR7/9 signaling strongly up-regulates expression of TACI, one of the receptors for BAFF and IFN-I stimulates BAFF expression by macrophages and dendritic cells (45). However, it is unclear whether BAFF-Tg mice exhibit the interferon signature.

Other single-gene defects affecting B-cell activation/maturation cause lupus-like disease. For example, deletion of the tyrosine kinase *Lyn* or over-expression of Bruton’s tyrosine kinase (*Btk*) is associated with lupus-like disease (46, 47). But these disorders have not been linked to dysregulated IFN-I production, suggesting that abnormalities in B-cell signaling may promote autoimmunity by IFN-I-independent mechanisms.

### “Interferonopathies” (TREM173, TREX1, MDA5, DNaseII, and Other Defects)

In addition to the endosomal nucleic acid sensors TLR3/TLR7/TLR8/TLR9, the cytoplasm contains sensors for DNA and RNA, the primary function of which is to detect microbial infection (48). These molecules also recognize endogenous nucleic acids, but in the normal state, self-DNA/RNA is sequestered away from the sensors and degraded before it can stimulate an IFN-I response (35, 49). However, when endogenous nucleic acid is inefficiently degraded, it can engage the sensors, resulting in interferon-mediated autoinflammatory diseases (16). In pediatric populations, screening for these disorders is carried out by testing for the interferon signature (17). Defects in genes involved in the STING pathway are an example. STING is an endoplasmic reticulum-associated protein that recognizes cytoplasmic DNA, resulting in IFN-I production via the activation of TBK1 and transcription factor IRF3 (19). Human gain of function mutations in *TREM173* (encodes STING) causes an IFN-I-mediated autoinflammatory syndrome characterized by cutaneous vasculopathy and pulmonary inflammation (19). However, ANAs are not prominent and lupus-like disease develops only rarely. Similarly, human loss-of-function mutations in *TREX1*, a negative regulator of STING (50), cause autoinflammatory disease manifested by severe neurological impairment (encephalopathy, calcification of the basal ganglia) caused by IFN-I production in the central nervous system (Aicardi–Goutieres syndrome) (51). They also are associated with familial chilblain lupus, an erythematous/violaceous rash typically involving the hands and/or feet but distinct from the classic SLE rashes. ANAs sometimes are produced, but not anti-dsDNA or anti-Sm/RNP, and typical clinical manifestations of SLE are rare. Murine *Trex1* deficiency is lethal, causing autoinflammatory disease with myocarditis and autoantibodies against cardiac muscle, but not the typical lupus autoantibodies. Interestingly, gain-of-function mutations of the cytoplasmic RNA sensor *MDA5* cause an IFN-I-mediated autoinflammatory syndrome similar to disorders of the STING/TREX1 axis (52). STING paradoxically suppresses lupus-like disease in MRL/*lpr* mice and in pristane-induced lupus. STING-deficient mice develop accelerated glomerulonephritis and mortality and STING-deficient MRL/*lpr* mice develop an interferon signature (18). The exacerbation of lupus may be related to low expression of A20, SOCS1, and SOCS3, which are negative regulators of TLR signaling, in STING-deficient mice.

Abnormal endosomal degradation of DNA released by apoptotic cells by DNase II also causes an inflammatory disorder (35). DNase II deficiency is embryonic lethal, but conditional knockout mutants develop IFN-I-mediated arthritis resembling rheumatoid arthritis with low levels of anti-dsDNA autoantibodies (10% of the level seen in MRL/*lpr* mice) (35). Although TLR independent, the disease is STING dependent (53).

The phenotypes of these interferonopathies illustrate an important point that while over-production of IFN-I (as indicated by the high interferon signature in such patients) is sufficient to cause autoinflammatory disease, it is not sufficient to cause lupus, possibly reflecting the synergistic effects of IFN-I over-production with delayed phagocytosis of apoptotic cells and abnormal B-cell activation/maturation. That possibility is supported by the phenotype of lupus-like disease in mice treated with pristane and by the fact that exogenous IFNα administration (via an adenoviral vector) is insufficient to induce lupus-like disease in non-autoimmune prone mice (22).

## Pristane-Induced Lupus

Pristane (2,6,10,14-tetramethylpentadecane) is an isoprenoid alkane found in plants, shark liver, and mineral oil, a byproduct of petroleum distillation. Intraperitoneal injection of pristane in non-autoimmune prone mice, such as B6 or BALB/c, causes a lupus-like syndrome with high levels of IgG anti-dsDNA, anti-Sm/RNP, anti-Su, anti-ribosomal P, and other lupus-related autoantibodies starting ~3 months after treatment (54). As in SLE patients, disease is more severe in females (55). The clinical manifestations exhibit strain-to-strain variability, and include immune complex-mediated glomerulonephritis (prominent in BALB/c and SJL mice), arthritis (BALB/c), anemia (most strains), and diffuse alveolar hemorrhage (DAH, B6, and B10 mice) (54, 56) (Table 1). Many of these manifestations are cytokine driven.

Like SLE patients, pristane-treated mice exhibit a strong IFN signature and mice deficient in the IFNAR do not develop autoantibodies or glomerulonephritis (54). In contrast, anemia in pristane-treated mice is dependent on TNFα and independent of IFN-I. The bone marrow of pristane-treated mice shows high levels of TNFα and a remarkable accumulation of apoptotic cells, abnormalities that are also present in the bone marrow of SLE patients (57). The arthritis in pristane-treated mice also is likely to be TNFα mediated as it is ameliorated by TNF inhibitor therapy (58). IFN-I and TNFα production is TLR7 dependent (57, 59). Nephritis and anemia are abolished in TLR7-deficient mice, but not in TLR9-deficient mice. Interestingly, the production of autoantibodies against RNA-protein antigens is TLR7 dependent, but so is production of anti-dsDNA autoantibodies. Pristane treatment causes the accumulation of dead cells in the bone marrow and other sites (57), which are likely to be the source of endogenous TLR7 ligands driving the disease. Pristane induces lupus in germ-free mice, emphasizing the probable role of endogenous rather than microbial TLR7 ligands in disease pathogenesis (60).

### IFN-I Production in Pristane-Induced Lupus

Although pristane-treated mice exhibit a robust IFN signature and autoantibody production and nephritis are abolished in IFNAR-deficient mice, the role of IFN-I in disease pathogenesis is incompletely understood. A population of inflammatory (Ly6C^hi^) monocytes produces most of the IFN-I in pristane-treated mice (61), although smaller amounts are produced by pDCs. Paradoxically, although wild-type B6 mice develop both autoantibodies and an interferon signature, Fas-deficient (B6/*lpr*) mice are refractory to the induction of autoantibodies by pristane despite exhibiting an interferon signature (62). The explanation is under investigation.

## Conclusion

Animal models have led to a clearer understanding of the role of IFN-I in the pathogenesis of lupus-like disease. One major effect of IFN-I over-production is enhanced TLR7 expression and TLR7 signaling, which may maintain IFN-I production as well as having intrinsic effects on B-cell activation and maturation. However, as illustrated by the interferonopathies, over-production of IFN-I, although pro-inflammatory, does not suffice to induce lupus-like disease. The pristane-lupus model suggests that the lupus phenotype depends on more than just IFN-I. TNFα influences the pathogenesis of some of the clinical manifestations (e.g., anemia, arthritis), whereas others are independent of both TNFα and IFN-I (e.g., DAH). By activating multiple inflammatory pathways as well as promoting the accumulation of apoptotic cells/endogenous TLR7 ligands and B-cell activation, the inducible pristane-lupus model may afford a window into the roles played by the multiple pathways shaping the widely variable phenotypes of SLE in different patients.

## Conflict of Interest Statement

The authors declare that the research was conducted in the absence of any commercial or financial relationships that could be construed as a potential conflict of interest.
